# Mood, Quality of Life, and Immune Fitness During the COVID-19 Pandemic of Young Adults in Germany

**DOI:** 10.3390/jcm13216487

**Published:** 2024-10-29

**Authors:** Pauline A. Hendriksen, Pantea Kiani, Anna Helin Koyun, Johan Garssen, Ann-Kathrin Stock, Joris C. Verster

**Affiliations:** 1 Division of Pharmacology, Utrecht Institute for Pharmaceutical Sciences, Utrecht University, Universiteitsweg 99, 3584 CG Utrecht, The Netherlands; p.a.hendriksen@students.uu.nl (P.A.H.); p.kiani@uu.nl (P.K.); j.garssen@uu.nl (J.G.); 2 Cognitive Neurophysiology, Department of Child and Adolescent Psychiatry, Faculty of Medicine, TU Dresden, D-01307 Dresden, Germany; annahelin.koyun@ukdd.de (A.H.K.); ann-kathrin.stock@ukdd.de (A.-K.S.); 3 Danone Global Research & Innovation Center, Uppsalalaan 12, 3584 CT Utrecht, The Netherlands; 4 Centre for Mental Health and Brain Sciences, Swinburne University, Melbourne, VIC 3122, Australia

**Keywords:** COVID-19, lockdown, mood, stress, quality of life, immune fitness

## Abstract

**Background**: The COVID-19 pandemic has profoundly affected young adults’ lives globally, including those in Germany. This study investigated mental health and quality of life during the pandemic, with a particular focus on mood. Immune fitness, the body’s capacity to respond to health challenges (such as infections) by activating an appropriate immune response, was assessed as a physical health indicator. **Methods**: Data were collected from 317 participants, aged 18 to 35, via an online survey conducted between November 2021 and March 2022. Participants included 103 men (32.5%) and 214 women (67.5%), with a mean age of 25.5 years (SD = 4.1). **Results**: Compared to pre-pandemic levels, significant declines in mood, quality of life, immune fitness, and sleep quality were observed during the lockdown periods of the COVID-19 pandemic (*p* < 0.0125). The most pronounced effects were observed during the second lockdown, with declines extending into the second no-lockdown period for fatigue, depression, happiness, optimism, and immune fitness (*p* < 0.0125). Significant sex differences were found for the magnitude of mood effects (anxiety, depression, stress). No significant differences were found according to age or occupational status (student vs. work). **Conclusions**: The COVID-19 pandemic and associated lockdown periods had a significant negative effect on the mood, immune fitness, and well-being of young adults living in Germany.

## 1. Introduction

The COVID-19 pandemic was declared a global health emergency by the World Health Organization on 11 March 2020, leading to unprecedented public health measures worldwide. In Germany, the government quickly imposed restrictions to mitigate the spread of the virus, resulting in widespread closures of schools and businesses and strict limitations on social interactions [1]. Essential public facilities, including supermarkets, hospitals, and pharmacies, remained operational under stringent hygiene protocols, which mandated the use of face masks [1].

The progression of the pandemic in Germany was characterized by alternating periods of strict lockdowns and gradual easing of restrictions. Young adults faced significant challenges during these transitions, including the shift to remote work and online learning, which reduced opportunities for social interaction considerably. The psychological impact of isolation and social distancing measures was profound [2,3].

Germany’s economy experienced significant disruptions, with many businesses, especially in non-essential sectors, facing closures, decreased revenues, layoffs, and permanent shutdowns [4,5]. The ‘Kurzarbeit’ policy, which allowed companies to reduce employees’ working hours while the government compensated a portion of their lost wages, helped mitigate some negative effects and stabilized the job market. Despite this, unemployment rates peaked at 4.1% in 2020, and the consumer price index (CPI) saw a notable increase in November 2021, exacerbating financial stress [6,7,8,9]. These economic pressures particularly affected those with pre-existing low incomes and precarious working conditions [10,11]. Economic difficulties and job instability, which are known to lower quality of life and to be linked to increased mental health issues [1,12,13], likely contributed to the observed decline in quality of life during the pandemic.

Globally, studies have highlighted the pandemic’s adverse effects on mental well-being, with similar trends observed in Germany. Reports indicate increases in loneliness, stress, depression, and anxiety, particularly among younger individuals and women, who were more vulnerable to pandemic-related stressors [14,15,16,17,18,19,20,21]. Fear of COVID-19 further exacerbated mental health issues for some individuals [15].

Addressing mental health complaints is critical due to their impact on daily functioning and productivity [22]. Prolonged stress can dysregulate the body’s stress response system, leading to overactivation of the sympathetic nervous system and increased cortisol levels, which can impair immune function and increase susceptibility to diseases, including COVID-19 [23,24]. Additionally, anxiety and depression can disrupt sleep patterns, further compromising the immune system [25,26].

The current study aimed to assess mood, immune fitness, and quality of life among young adults in Germany before and during the COVID-19 pandemic. Specifically, it examined changes in mood and quality of life over different phases of the pandemic, as well as potential sex and age differences in these outcomes. Immune fitness is an important determinant of health and disease and refers to the body’s capacity to respond to health challenges (such as infections) by activating an appropriate immune response [27]. Therefore, in this study, immune fitness was assessed as a physical health indicator. Based on the results of previous studies conducted in the Netherlands, Argentina, and Türkiye [28,29] using the same study design [30,31], it was hypothesized that the negative effects on mood, immune fitness, and quality of life were more pronounced during lockdown periods than during no-lockdown periods (including the pre-pandemic period).

## 2. Methods

An online survey was conducted between mid-November 2021 and the end of March 2022. Potential participants were invited to participate in this study via university e-mail and printed flyers, mainly distributed in the city of Dresden. Eligible participants were those residing in Germany and aged between 18 and 35 years. There were no other inclusion or exclusion criteria.

This study was reviewed and approved by the Ethics Committee of the Medical Faculty of TU Dresden (approval code: SR-EK-8012020, date of approval: 27 September 2021). All participants provided electronic informed consent, and this study was conducted in accordance with the Declaration of Helsinki and its latest amendments. Participants could enter a prize draw with the chance to win one of four EUR 25 Amazon gift vouchers. 

The survey was designed using LimeSurvey (open-source survey tool; Version 5.0.11+210727, Hamburg, Germany: LimeSurvey GmbH). Participants could complete the survey in German language or English (in case German was not their primary language). The survey, raw data, and detailed description of the study methodology have been published elsewhere [32].

Demographic data included age, sex (male or female), living situation (alone or with family or friends), and occupational status (student or employed). Mood was assessed with single-item rating scales ranging from 0 (absent) to 10 (extreme) and included stress, anxiety, depression, fatigue, loneliness, optimism, and happiness [33,34]. Quality of life [35], sleep quality [36], and immune fitness [27] were rated on scales, ranging from 0 (very poor) to 10 (excellent). Assessments were made for five periods: (1) ‘BP’ (the period before the COVID-19 pandemic), (2) ‘L1’ (the first lockdown period, March–May 2020), (3) ‘NL1’ (the first no-lockdown period, summer 2020), (4) ‘L2’ (the second lockdown, November 2020 to May 2021), and (5) ‘NL2’ (the second no-lockdown period, summer 2021).

Statistical analyses were conducted with SPSS (IBM Corp. Released 2013. IBM SPSS Statistics for Windows, Version 29.0. Armonk, NY, USA: IBM Corp.). Mean and standard deviation (SD) were computed for all variables. Since the data were not normally distributed, nonparametric tests were applied. Within-subject comparisons of the five time points were conducted with the Related-Sample Friedman Two-Way Analysis of Variance by Ranks test. A Bonferroni correction was applied, so that differences were considered significant if *p* < 0.0125. Between-group comparisons were conducted with the Independent Samples Mann–Whitney U Tests. Groups were formed according to sex (males versus females), age (18–24 years old versus 25–35 years old), occupation (student versus employed), and living situation (alone versus with family or friends).

## 3. Results

**Figure 1:**
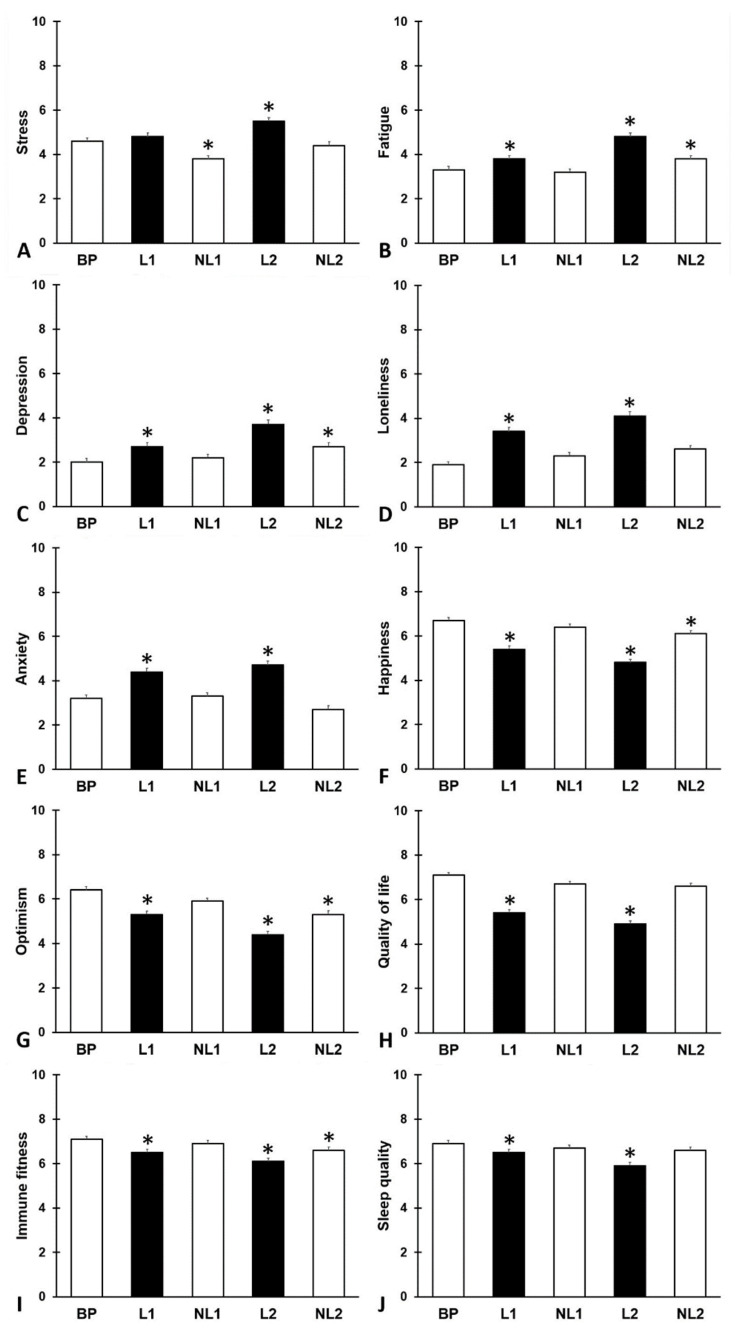
Mood assessments. Mean and standard error are shown for (**A**) stress, (**B**) fatigue, (**C**) depression, (**D**) loneliness, (**E**) anxiety, (**F**) happiness, (**G**) optimism, (**H**) quality of life, (**I**) immune fitness, and (**J**) sleep quality. Abbreviations: BP = before the pandemic, L1 = lockdown 1, NL1 = no lockdown 1, L2 = lockdown 2, NL2 = no lockdown 2, COVID-19 = coronavirus disease 2019. Significant differences from ‘before the pandemic’ (*p* < 0.0125) are indicated by *.

A total of N = 317 participants (103 men and 214 women) completed the survey. Their mean (SD) age was 25.5 (4.1) years old. Of the sample, N = 207 participants were students and N = 110 had a job during the COVID-19 pandemic. The study outcomes are summarized in Figure 1 and Table 1. 

Compared to BP, mood, quality of life, immune fitness, and sleep quality were significantly poorer during the two lockdown periods (L1 and L2). The effects were most pronounced during L2, with significant declines in fatigue, happiness, optimism, and immune fitness extending into NL2 compared to BP. 

**Figure 2:**
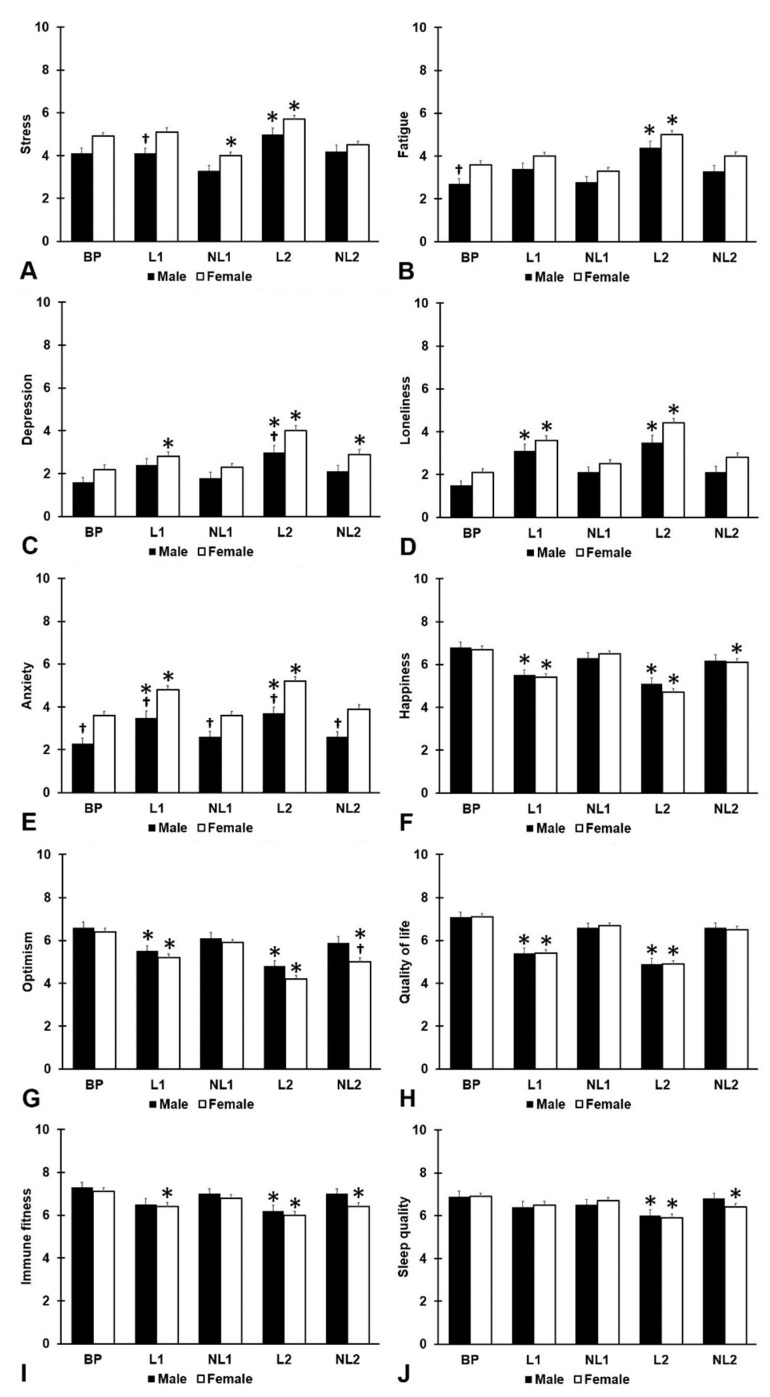
Mood assessments according to sex. Mean and standard error are shown for (**A**) stress, (**B**) fatigue, (**C**) depression, (**D**) loneliness, (**E**) anxiety, (**F**) happiness, (**G**) optimism, (**H**) quality of life, (**I**) immune fitness, and (**J**) sleep quality. Abbreviations: BP = before the pandemic, L1 = lockdown 1, NL1 = no lockdown 1, L2 = lockdown 2, NL2 = no lockdown 2, COVID-19 = coronavirus disease 2019. Significant differences from ‘before the pandemic’ (*p* < 0.0125) are indicated by *. Significant differences between men and women (*p* < 0.010, after Bonferroni’s correction) are indicated by †.

The study outcomes according to sex are summarized in Figure 2 and Table 2. Both males (N = 103, 32.5%) and females (N = 214, 67.5%) reported that compared to BP, mood, quality of life, immune fitness, and sleep quality were significantly poorer during the two lockdown periods (L1 and L2), with most pronounced effects observed during L2. Significant sex differences were found for stress, depression, anxiety, and optimism. During L1, the reported stress level of females was significantly higher than that of males (*p* = 0.003). Anxiety levels of females were significantly higher than those of males for all assessment periods, including BP (all *p* < 0.001). Depression levels of females during L2 were significantly higher compared to males (*p* = 0.010), and females were significantly less optimistic than males during NL2 (*p* = 0.011).

No significant differences according to age were found (see Table 3). The 18–24-year-old group (N = 146, 46.1%) and the 25–35-year-old group (N = 171, 53.9%) reported similar patterns of poorer mood, immune fitness, and sleep quality during the lockdown periods. 

Analyses according to occupation showed a similar outcome pattern (see Table 4). The only difference between students (N = 207, 65.3%) and those with a job (N = 110, 34.7%) was increased loneliness levels among students both before the pandemic (*p* = 0.013) and at L1 (*p* < 0.001), NL1 (*p* = 0.032), L2 (*p* = 0.001), and NL2 (*p* = 0.011). N = 98 (30.9%) participants were living alone, and N = 219 (69.1%) were living together with friends or family during the COVID-19 pandemic.

No significant differences were found according to living situation (see Table 5). For both groups, comparable patterns of poorer mood, immune fitness, and sleep quality were reported for the two lockdown periods relative to BP, with the most pronounced effects observed during L2.

## 4. Discussion

This study examined the impact of the COVID-19 pandemic on the mental well-being of young adults in Germany. The findings show significant declines in mood, quality of life, immune fitness, and sleep quality during the two lockdown periods, L1 and L2, with the most pronounced effects noted during L2. These outcomes align with prior research and underscore the need for targeted mental health support during such crises [16,27,28,37,38,39,40,41].

The observed decline in mood may be partially attributable to disruptions to daily routines, which may have exacerbated feelings of isolation and loneliness, especially among university students. The shift to remote learning and the absence of on-campus activities led to a significant sense of loss and isolation [42,43,44]. The closure of social outlets confined many young adults to their homes, intensifying loneliness due to the lack of customary social interactions and support networks [45,46,47]. Additionally, the transition to remote work and online learning required swift adaptation, likely contributing to declines in mood [48]. This shift may have intensified feelings of isolation and loneliness, particularly for those relying heavily on social interactions for support [49].

Uncertainties about the crisis duration and severity, along with fear of infection, likely heightened stress and anxiety levels [16,20]. COVID-19-related fear also correlated with increased depression symptoms, deteriorating health status, distress, and generalized anxiety symptoms [15,50]. The data reveal a significant increase in loneliness during the lockdown periods, aligning with international reports [39,40,41]. While research on the prevalence of loneliness among young Germans is mixed [51,52], the findings presented here add to the literature on its impact during the lockdowns. Some individuals found virtual means to connect, partially mitigating isolation [53].

Financial concerns significantly contributed to the decline in mood among young adults in Germany during the pandemic. Despite governmental efforts, financial hardships persisted [8,54]. The shutdown of businesses, particularly in the service industry, led to job losses and financial uncertainty [8]. Economic instability and reduced career prospects further fueled financial anxiety, placing considerable stress on young adults and students [8,11,55]. Furthermore, financial worries can exacerbate pre-existing mental health issues potentially triggered by the pandemic [55,56,57], compounding the observed decline in mood. Germany’s strong social market economy, despite its robust welfare systems, was strained during the pandemic. Support mechanisms, though helpful, could not fully mitigate economic anxieties, especially among students and young adults in precarious unemployment [5,6,7]. The initial public support for lockdown measures in Germany, which declined over time, correlates with the increase in anxiety and depression among young adults [5].

The current study showed a decline in both mood and sleep quality, likely due to lifestyle changes following the onset of the COVID-19 pandemic. Jabakhanji et al. [58] attribute the reduction in sleep quality to changes in lifestyles and worse mental health, highlighting the close relationship between sleep quality and mood. Lower sleep quality, in turn, can decrease immune fitness and increase susceptibility to infection [59]. 

Furthermore, movement restrictions and the closure of recreational facilities significantly reduced physical activity levels, leading to a more sedentary lifestyle [60]. Reduced physical activity can negatively impact sleep quality, mood, and quality of life [61,62,63,64]. Physical activity has been shown to improve depressive and anxiety symptoms, and those less active are more likely to experience loneliness [61,63,65,66]. Herbolsheimer et al. [67] reported significant reductions in physical activity and increased sedentary time among German participants during the pandemic, with over a third failing to meet the WHO’s physical activity guidelines [68]. Additionally, depressive symptoms were associated with increased sedentary behavior, particularly among young adults, aligning with findings linking reduced physical activity to greater distress and anxiety symptoms [67,69].

The significant decreases in immune fitness may be influenced by various stressors prevalent during the pandemic. Increased stress and anxiety levels, declines in sleep quality, reduced physical activity, and social isolation can all negatively impact immune functioning [70]. Stress, characterized by the release of hormones such as cortisol, can significantly suppress immune functioning, thereby increasing susceptibility to infection. If stress becomes chronic, it can lead to glucocorticoid (receptor-) resistance, hindering the hypothalamic–pituitary–adrenal (HPA) axis’s ability to regulate pro-inflammatory cytokines effectively, resulting in heightened levels of inflammation [71]. Social isolation can exacerbate distress, further compromising immune function [72]. Additionally, declines in sleep quality and reduced physical activity are known to weaken immune function [73]. Poor sleep quality can disrupt circadian rhythms and suppress immune responses, while reduced physical activity can weaken immune defenses [74]. Regular exercise offers significant benefits for the immune systems in terms of susceptibility, progression, and outcome of infections like COVID-19 [75]. The combined effect of these factors can contribute to weakened immune functioning and a reduction in self-reported immune fitness. As shown previously, lower immune fitness makes individuals more vulnerable to illnesses [76], including the presence and severity of COVID-19 symptoms [77].

Several sex differences were observed. Women consistently reported higher anxiety levels across all time periods, aligning with existing research [78,79,80]. This observation may arise from sex differences in factors such as socialization, psychosocial traits, biological distinctions in brain structures, and hormonal fluctuations. Neurobiological studies have identified differences in amygdala volume and cerebral blood flow, as well as serotonin transporter gene expression, which may increase women’s susceptibility to anxiety [80,81,82,83]. Additionally, women may be more likely to seek help and disclose mental health concerns, while men might underreport symptoms due to societal masculinity norms [84].

The observed increase in depression levels among women during the second lockdown may be related to occupational representation, caregiving responsibilities, and the economic impact of the COVID-19 pandemic [85]. Women in Germany are overrepresented in sectors like healthcare, education, and retail, which were heavily affected by lockdown measures, leading to decreased work-related social interactions, economic instability, and stress [86,87,88]. Additionally, the closure of schools and childcare services increased caregiving responsibilities, adding stress as women balanced childcare, household duties, and work [85,89]. Many women reduced their working hours to manage increased caregiving responsibilities, as they were often the lower-earning parents, making it economically sensible for them to take on more caregiving responsibilities [14]. The cumulative effects of prolonged stress and reduced social interaction causing feelings of loneliness likely contributed to the observed increase in depression during the second lockdown [3,88,90,91,92].

This study has several limitations that should be considered when interpreting its findings. The recruitment strategy, which utilized university emails and printed flyers in Dresden, might lead to a sampling bias favoring more educated individuals from urban areas, thereby limiting the generalizability of the results to other age groups or populations. Additionally, this study relied on retrospective self-report measures to assess mood, quality of life, and immune fitness, which are vulnerable to recall bias and social desirability bias. The latter could have introduced inaccuracies in the provided answers to the survey. Important variables such as pre-existing mental health conditions, coping strategies, social support networks, and access to healthcare services, which could influence young adults’ well-being, were not assessed, limiting our ability to comprehensively understand the factors affecting their overall well-being. The cross-sectional study design also precludes the establishment of causal relationships between variables. Moreover, the survey’s availability solely in English and German may have introduced language and cultural biases, potentially excluding non-speakers. Future studies should address these limitations by exploring the long-term effects of the COVID-19 pandemic on mental health outcomes, evaluating intervention strategies, and examining how social factors contribute to mental health disparities to enhance our understanding of how crises impact mental well-being and inform effective responses.

## 5. Conclusions

This study of young adults in Germany during the COVID-19 pandemic highlights the profound negative impacts of the pandemic and associated lockdowns on mood, quality of life, immune fitness, and sleep quality. Our results reveal significant sex differences, with women consistently reporting higher levels of anxiety, more stress during the first lockdown, and higher depression scores during the second lockdown. The findings underscore the necessity for ongoing mental health support and the integration of these considerations into public health planning and response.

## Figures and Tables

**Table 1:** Study outcomes.

Time Period	BP	L1	NL1	L2	NL2	Overall	BP vs. L1	BP vs. NL1	BP vs. L2	BP vs. NL2
Stress	4.6 (2.4)	4.8 (2.7)	3.8 (3.7)	5.5 (2.5)	4.4 (2.6)	<0.001 *	0.374	<0.001 *	<0.001 *	0.198
Fatigue	3.3 (2.5)	3.8 (2.6)	3.2 (2.4)	4.8 (2.8)	3.8 (2.6)	<0.001 *	0.003 *	0.226	<0.001 *	0.002 *
Depression	2.0 (2.6)	2.7 (2.9)	2.2 (2.5)	3.7 (3.1)	2.7 (3.0)	<0.001 *	<0.001 *	0.594	<0.001 *	0.002 *
Loneliness	1.9 (2.3)	3.4 (3.1)	2.3 (2.6)	4.1 (3.2)	2.6 (2.8)	<0.001 *	<0.001 *	0.312	<0.001 *	0.013
Anxiety	3.2 (2.6)	4.4 (2.9)	3.3 (2.6)	4.7 (3.0)	3.5 (2.7)	<0.001 *	<0.001 *	0.586	<0.001 *	0.095
Happiness	6.7 (2.3)	5.4 (2.4)	6.4 (2.2)	4.8 (2.5)	6.1 (2.5)	<0.001 *	<0.001 *	0.060	<0.001 *	0.003 *
Optimism	6.4 (2.5)	5.3 (2.4)	5.9 (2.3)	4.4 (2.5)	5.3 (2.7)	<0.001 *	<0.001 *	0.019	<0.001 *	<0.001 *
Quality of life	7.1 (2.0)	5.4 (2.3)	6.7 (1.8)	4.9 (2.4)	6.6 (2.1)	<0.001 *	<0.001 *	0.259	<0.001 *	0.079
Immune fitness	7.1 (2.3)	6.5 (2.5)	6.9 (2.3)	6.1 (2.5)	6.6 (2.4)	<0.001 *	0.001 *	0.221	<0.001 *	0.002 *
Sleep quality	6.9 (2.2)	6.5 (2.3)	6.7 (2.2)	5.9 (2.4)	6.6 (2.3)	<0.001 *	0.004 *	0.246	<0.001 *	0.014

Mean and standard deviation (SD) are shown. Significant differences between before the pandemic (BP) and the other time periods are indicated by *. Pairwise comparisons were computed if the main effect was significant (*p* < 0.05) and considered significant if *p* < 0.0125 after Bonferroni’s correction. Abbreviations: BP = before the pandemic, L1 = lockdown 1, NL1 = no lockdown 1, L2 = lockdown 2, NL2 = no lockdown 2, COVID-19 = coronavirus disease 2019.

**Table 2:** Study outcomes according to sex.

Time Period	BP		L1		NL1		L2		NL2	
Sex	Men	Women	Men	Women	Men	Women	Men	Women	Men	Women
Stress	4.1 (2.5)	4.9 (2.3)	4.1 (2.5)	5.1 (2.7) †	3.3 (2.2)	4.0 (2.4) *	5.0 (2.7) *	5.7 (2.4) *	4.2 (2.8)	4.5 (2.6)
Fatigue	2.7 (2.4)	4.0 (2.5) †	3.4 (2.6)	4.0 (2.5)	2.8 (2.4)	3.3 (2.4)	4.4 (2.9) *	5.0 (2.7) *	3.3 (2.5)	4.0 (2.6)
Depression	1.6 (2.1)	2.2 (2.7)	2.4 (2.9)	2.8 (2.9) *	1.8 (2.5)	2.3 (2.6)	3.0 (3.1) *	4.0 (3.1) †*	2.1 (2.7)	2.9 (3.0) *
Loneliness	1.5 (2.0)	2.1 (2.4)	3.1 (3.1) *	3.6 (3.0) *	2.1 (2.6)	2.5 (2.6)	3.5 (3.2) *	4.4 (3.2) *	2.1 (2.7)	2.8 (2.8)
Anxiety	2.3 (2.4)	3.6 (2.6) †	3.5 (2.9) *	4.8 (2.8) †*	2.6 (2.5)	3.6 (2.6) †	3.7 (2.9) *	5.2 (3.0) †*	2.6 (2.3)	3.9 (2.8) †
Happiness	6.8 (2.3)	6.7 (2.2)	5.5 (2.6) *	5.4 (2.4) *	6.3 (2.5)	6.5 (2.0)	5.1 (2.6) *	4.7 (2.4) *	6.2 (2.6)	6.1 (2.4) *
Optimism	6.6 (2.5)	6.4 (2.4)	5.5 (2.5) *	5.2 (2.4) *	6.1 (2.4)	5.9 (2.2)	4.8 (2.5) *	4.2 (2.5) *	5.9 (2.7)	5.0 (2.7) †*
Quality of life	7.1 (2.1)	7.1 (2.0)	5.4 (2.4) *	5.4 (2.3) *	6.6 (1.9)	6.7 (1.8)	4.9 (2.7) *	4.9 (2.2) *	6.6 (2.1)	6.5 (2.1)
Immune fitness	7.3 (2.2)	7.1 (2.3)	6.5 (2.6)	6.4 (2.5) *	7.0 (2.3)	6.8 (2.2)	6.2 (2.6) *	6.0 (2.5) *	7.0 (2.2)	6.4 (2.4) *
Sleep quality	6.9 (2.4)	6.9 (2.2)	6.4 (2.4)	6.5 (2.2)	6.5 (2.4)	6.7 (2.0)	6.0 (2.6) *	5.9 (2.3) *	6.8 (2.3)	6.6 (2.3) *

Mean and standard deviation (SD) are shown. Significant differences between men and women (*p* < 0.0125 after Bonferroni’s correction) are indicated by †. Pairwise comparisons were computed if the main effect was significant (*p* < 0.05). Significant differences between BP and the other time periods (*p* < 0.0125 after Bonferroni’s correction) are indicated by *. Abbreviations: BP = before the pandemic, L1 = lockdown 1, NL1 = no lockdown 1, L2 = lockdown 2, NL2 = no lockdown 2, COVID-19 = coronavirus disease 2019.

**Table 3:** Study outcomes according to age group.

Time Period	BP		L1		NL1		L2		NL2	
Age (years)	18–24	25–35	18–24	25–35	18–24	25–35	18–24	25–35	18–24	25–35
Stress	4.8 (2.3)	4.5 (2.5)	4.5 (2.6)	5.0 (2.8)	3.6 (2.2) *	3.9 (2.5)	5.5 (2.4) *	5.5 (2.7) *	4.3 (2.5)	4.5 (2.7)
Fatigue	3.6 (2.4)	3.0 (2.6)	3.9 (2.5)	3.7 (2.6)	3.3 (2.4)	3.0 (2.3)	5.0 (2.7) *	4.7 (2.9) *	3.9 (2.5)	3.6 (2.7) *
Depression	1.9 (2.7)	2.1 (2.4)	2.5 (2.9)	2.9 (2.9) *	1.9 (2.5)	2.4 (2.6)	3.6 (3.2) *	3.7 (3.1) *	2.5 (3.1)	2.8 (2.9) *
Loneliness	2.1 (2.3)	1.7 (2.3)	3.5 (3.0) *	3.4 (3.1) *	2.3 (2.5)	2.3 (2.7)	4.2 (3.2) *	4.0 (3.2) *	2.5 (2.7)	2.7 (2.9) *
Anxiety	3.1 (2.5)	3.3 (2.7)	4.0 (2.7) *	4.7 (3.0) *	3.2 (2.4)	3.4 (2.8)	4.7 (3.0) *	4.8 (3.1) *	3.3 (2.6)	3.6 (2.8)
Happiness	6.7 (2.3)	6.8 (2.2)	5.6 (2.3) *	5.3 (2.5) *	6.6 (2.1)	6.2 (2.2)	4.6 (2.4) *	4.9 (2.6) *	6.3 (2.5)	5.9 (2.4) *
Optimism	6.4 (2.4)	6.5 (2.5)	5.4 (2.3) *	5.2 (2.5) *	6.0 (2.2)	5.9 (2.3)	4.3 (2.5) *	4.5 (2.5) *	5.4 (2.7) *	5.2 (2.8) *
Quality of life	6.9 (2.1)	7.2 (2.0)	5.6 (2.2) *	5.2 (2.4) *	6.8 (1.9)	6.6 (1.8)	4.8 (2.3) *	5.0 (2.5) *	6.7 (2.0)	6.5 (2.2)
Immune fitness	7.1 (2.2)	7.2 (2.4)	6.6 (2.5)	6.4 (2.6) *	6.8 (2.2)	6.9 (2.3)	6.1 (2.4) *	6.1 (2.6) *	6.6 (2.3)	6.6 (2.5)
Sleep quality	6.8 (2.3)	7.0 (2.2)	6.7 (2.2)	6.3 (2.4) *	6.6 (2.1)	6.7 (2.2)	5.8 (2.3) *	6.0 (2.5) *	6.5 (2.2)	6.6 (2.4)

Mean and standard deviation (SD) are shown. No significant differences between the 18–24-year-old group and the 25–35-year-old group (*p* < 0.0125 after Bonferroni’s correction) were found. Pairwise comparisons were computed if the main effect was significant (*p* < 0.05). Significant differences between BP and the other time periods (*p* < 0.0125 after Bonferroni’s correction) are indicated by *. Abbreviations: BP = before the pandemic, L1 = lockdown 1, NL1 = no lockdown 1, L2 = lockdown 2, NL2 = no lockdown 2, COVID-19 = coronavirus disease 2019.

**Table 4:** Study outcomes according to occupation.

Time Period	BP		L1		NL1		L2		NL2	
Occupation	Student	Job	Student	Job	Student	Job	Student	Job	Student	Job
Stress	4.6 (2.3)	4.7 (2.6)	4.9 (2.7) *	4.6 (2.7)	3.8 (2.3) *	3.8 (2.4) *	5.5 (2.5) *	5.4 (2.6) *	4.4 (2.6)	4.4 (2.8)
Fatigue	3.2 (2.4)	3.4 (2.7)	3.9 (2.6) *	3.6 (2.5)	3.2 (2.4)	3.2 (2.4)	5.0 (2.7) *	4.4 (2.9) *	3.8 (2.6) *	3.7 (2.7)
Depression	1.9 (2.5)	2.2 (2.7)	2.8 (2.9) *	2.5 (2.9)	2.3 (2.6)	2.0 (2.5)	3.9 (3.2) *	3.3 (3.0) *	2.8 (3.0) *	2.5 (2.9)
Loneliness	2.1 (2.3)	1.6 (2.3)	3.9 (3.1) *	2.6 (2.8) *†	2.5 (2.6)	1.9 (2.5)	4.6 (3.3) *	3.2 (3.0) *†	2.9 (2.9)	2.1 (2.6) †
Anxiety	3.1 (2.5)	3.3 (2.8)	4.5 (2.8) *	4.3 (3.0) *	3.4 (2.6)	3.1 (2.5)	4.8 (3.1) *	4.7 (2.9) *	3.7 (2.7) *	3.1 (2.8)
Happiness	6.8 (2.2)	6.7 (2.3)	5.4 (2.5) *	5.5 (2.4) *	6.4 (2.2)	6.5 (2.1)	4.7 (2.5) *	5.1 (2.5) *	6.0 (2.6) *	6.2 (2.3)
Optimism	6.5 (2.4)	6.3 (2.7)	5.1 (2.4) *	5.6 (2.3) *	6.0 (2.2)	5.8 (2.4)	4.3 (2.6) *	4.5 (2.4) *	5.4 (2.7) *	5.1 (2.9) *
Quality of life	7.1 (2.1)	7.0 (2.0)	5.4 (2.3) *	5.4 (2.3) *	6.7 (1.8)	6.6 (1.8)	5.0 (2.4) *	4.7 (2.3) *	6.6 (2.2)	6.4 (1.9)
Immune fitness	7.3 (2.1)	6.8 (2.5)	6.7 (2.3)	6.0 (2.8) *	7.0 (2.0)	6.5 (2.6)	6.3 (2.4) *	5.7 (2.7) *	6.7 (2.3) *	6.4 (2.6)
Sleep quality	7.1 (2.2)	6.7 (2.3)	6.5 (2.4)	6.4 (2.2)	6.7 (2.2)	6.5 (2.1)	6.0 (2.5) *	5.8 (2.3) *	6.6 (2.3)	6.4 (2.3)

Mean and standard deviation (SD) are shown. Significant differences between students and those with a job (*p* < 0.0125 after Bonferroni’s correction) are indicated by †. Pairwise comparisons were computed if the main effect was significant (*p* < 0.05). Significant differences between BP and the other time periods (*p* < 0.0125 after Bonferroni’s correction) are indicated by *. Abbreviations: BP = before the pandemic, L1 = lockdown 1, NL1 = no lockdown 1, L2 = lockdown 2, NL2 = no lockdown 2, COVID-19 = coronavirus disease 2019.

**Table 5:** Study outcomes according to living situation.

Time Period	BP		L1		NL1		L2		NL2	
Living Situation	Alone	Together	Alone	Together	Alone	Together	Alone	Together	Alone	Together
Stress	4.8 (2.7)	4.6 (2.3)	4.8 (2.8)	4.8 (2.6)	3.7 (2.5) *	3.8 (2.3) *	5.8 (2.8) *	5.3 (2.4) *	4.2 (2.8)	4.5 (2.5)
Fatigue	3.2 (2.7)	3.3 (2.4)	3.5 (2.7)	3.9 (2.5) *	2.9 (2.5)	3.3 (2.3)	4.7 (2.9) *	4.9 (2.7) *	3.7 (2.6)	3.8 (2.6)
Depression	2.1 (2.6)	2.0 (2.5)	2.7 (3.0)	2.7 (2.8) *	2.5 (2.8)	2.0 (2.4)	4.1 (3.2) *	3.5 (3.1) *	2.8 (2.9)	2.6 (3.0) *
Loneliness	2.0 (2.4)	1.8 (2.2)	3.5 (3.1) *	3.4 (3.0) *	2.6 (2.9)	2.2 (2.4)	4.6 (3.3) *	3.9 (3.2) *	2.9 (2.9)	2.5 (2.8)
Anxiety	3.3 (2.9)	3.1 (2.4)	4.5 (3.1) *	4.4 (2.8) *	3.3 (2.9)	3.3 (2.4)	4.9 (3.3) *	4.7 (2.9) *	3.3 (2.9)	3.6 (2.7)
Happiness	6.6 (2.4)	6.8 (2.2)	5.3 (2.5) *	5.5 (2.4) *	6.1 (2.6)	6.6 (1.9)	4.5 (2.8) *	5.0 (2.3) *	6.0 (2.7)	6.2 (2.3) *
Optimism	6.3 (2.8)	6.5 (2.3)	5.0 (2.7) *	5.4 (2.2) *	5.6 (2.6)	6.1 (2.1)	4.0 (2.7) *	4.5 (2.4) *	5.4 (2.7) *	5.3 (2.8) *
Quality of life	7.0 (2.1)	7.1 (2.0)	5.5 (2.4) *	5.3 (2.2) *	6.6 (2.0)	6.7 (1.7)	4.8 (2.6) *	4.9 (2.3) *	6.5 (2.3)	6.6 (2.0)
Immune fitness	6.9 (2.4)	7.2 (2.2)	6.2 (2.5)	6.6 (2.5) *	6.7 (2.4)	7.0 (2.2)	5.9 (2.6) *	6.2 (2.5) *	6.4 (2.6)	6.7 (2.3) *
Sleep quality	6.9 (2.3)	6.9 (2.2)	6.5 (2.4)	6.5 (2.3)	6.7 (2.3)	6.7 (2.1)	5.5 (2.6) *	6.1 (2.3) *	6.3 (2.5)	6.7 (2.2)

Mean and standard deviation (SD) are shown. No significant differences between those who live alone and those who live together with family or friends (*p* < 0.0125 after Bonferroni’s correction) were found. Pairwise comparisons were computed if the main effect was significant (*p* < 0.05). Significant differences between BP and the other time periods (*p* < 0.0125 after Bonferroni’s correction) are indicated by *. Abbreviations: BP = before the pandemic, L1 = lockdown 1, NL1 = no lockdown 1, L2 = lockdown 2, NL2 = no lockdown 2, COVID-19 = coronavirus disease 2019.

## Data Availability

The dataset is published open access in the MDPI journal Data and is available online as a supplement to Koyun et al. [32].
